# P-1235. Should the Line for Linezolid be Less Zolid? Assessing the Safety and Tolerability of Linezolid for Durations of Therapy Greater than 14 Days

**DOI:** 10.1093/ofid/ofaf695.1427

**Published:** 2026-01-11

**Authors:** Jessica Lai, Alexander R Cain, Emily L Heil, Jonathan S Lapin, Sara Lee, Mandee Booth, Ronald Rabinowitz, Hyunuk Seung

**Affiliations:** University of Maryland Medical Center, Baltimore, MD; University of Maryland Medical Center, Baltimore, MD; University of Maryland School of Pharmacy, Baltimore, MD; University of Maryland Medical Center, Baltimore, MD; University of Maryland Medical Center, Baltimore, MD; University of Maryland School of Pharmacy, Baltimore, MD; University of Maryland School of Medicine, baltimore, Maryland; University of Maryland School of Pharmacy, Baltimore, MD

## Abstract

**Background:**

There is hesitance to use linezolid for durations past 14 days due to adverse drug events (ADEs), such as myelosuppression and neuropathy. This study evaluated the incidence of and patient characteristics associated with ADEs.

**Methods:**

This was a single-center, retrospective, observational cohort study. Adult patients prescribed linezolid for greater than 14 consecutive days at the time of discharge from 1/1/2020 to 7/31/2024 were included. Patients with outpatient laboratory results for hemoglobin, white blood cell, and platelet count were assessed for myelosuppression. Development of neuropathies were assessed via documentation in the medical record. Patient demographics, such as age, weight, creatinine clearance (CrCl) and treatment-related information were evaluated. The primary outcome was assessed using descriptive statistics to describe incidence of ADEs. Secondary outcomes were assessed using multivariate logistic regression analysis to determine patient characteristics associated with ADEs and rates of therapy discontinuation due to ADEs.

**Results:**

Of 311 screened patients, 159 met inclusion criteria. A total of 143 (89.9%) patients completed prescribed therapy while 16 (10.1%) discontinued early. Patients with older age, lower baseline platelet count, lower baseline CrCl, and myelosuppression were more likely to stop treatment early. Neuropathy was documented in 6 patients (3.8%). Of 118 patients assessed for myelosuppression, 26 cases occurred in 23 patients (19.5%). Thrombocytopenia occurred in 9 patients (7.6%). Patients who developed myelosuppression were more likely to be older, have a lower baseline creatinine clearance, and discontinue therapy early. After multivariate regression, only lower baseline CrCl was significantly associated with increased myelosuppression risk.

**Conclusion:**

Overall, the use of linezolid for durations greater than 14 days was well tolerated as evident by the treatment completion rate of nearly 90% despite a myelosuppression incidence of 19.5%. Our findings suggest an increased risk of myelosuppression in patients with reduced renal function. When considering patients for prolonged durations of therapy with linezolid, caution should be taken in patients with reduced renal function and increased age.

**Disclosures:**

Emily L. Heil, PharmD, MS, Wolters Kluwer: Advisor/Consultant

